# Clinical features and in-hospital mortality predictors of concurrent cardio-cerebral infarction: insights from a dual-center retrospective study

**DOI:** 10.3389/fneur.2024.1465144

**Published:** 2024-10-08

**Authors:** Weiwei Gao, Lingfeng Yu, Shouyue Jin, Lijuan Cai, Jingjing Fang, Xiaoqian Wang, Qingwei Yang, Xingyu Chen, Tao Ye, Renjing Zhu

**Affiliations:** ^1^Department of Neurology, Zhongshan Hospital of Xiamen University, School of Medicine, Xiamen University, Xiamen, China; ^2^Xiamen Cardiovascular Hospital of Xiamen University, School of Medicine, Xiamen University, Xiamen, China; ^3^Department of Cardiovascular, West China Xiamen Hospital of Sichuan University, Xiamen, China

**Keywords:** cardio-cerebral infarction, in-hospital mortality, neutrophil-to-lymphocyte ratio, albumin, N-terminal pro-B-type natriuretic peptide

## Abstract

**Objective:**

This study aimed to enhance the understanding of cardio-cerebral infarction (CCI) clinical features and identify key prognostic factors, thereby providing an empirical foundation for advancing prevention and treatment strategies and ultimately improving clinical outcomes for CCI patients.

**Methods:**

We retrospectively analyzed 17,645 AIS and 7,584 AMI patients admitted to two hospitals from 2014 to 2023. Univariate analysis, Spearman correlation, and multivariate logistic regression were performed to identify independent risk factors. Receiver operating characteristic (ROC) curves were used to determine optimal cutoff values.

**Results:**

This study enrolled 85 patients with CCI, representing an overall CCI incidence of approximately 0.34%. Males comprised 64.71% of the cohort. ST-segment elevation myocardial infarction and cardiogenic cerebral infarction were the most predominant subtypes. The in-hospital mortality rate was 30.59%, with 65.38% of deaths attributed to cardiac causes. Multivariate logistic regression analysis identified three independent risk factors for in-hospital mortality: elevated neutrophil-to-lymphocyte ratio (NLR), decreased serum albumin, and increased peak N-terminal pro-B-type natriuretic peptide levels (NT-proBNP). ROC curve analysis demonstrated that the area under the curve (AUC) for the NLR, albumin concentration and peak NT-proBNP concentration were 0.863, 0.723, and 0.824, respectively. The optimal cutoff values were 6.914 for NLR, 33.80 g/L for albumin, and 9474.50 pg/mL for peak NT-proBNP. The AUC of the combined diagnostic model reached 0.959, significantly outperforming the individual indicators.

**Conclusion:**

Elevated NLR, decreased serum albumin, and increased peak NT-proBNP levels independently predict in-hospital mortality in CCI patients. Combining these biomarkers enhances predictive capability for adverse outcomes.

## 1 Introduction

Acute myocardial infarction (AMI) and acute ischemic stroke (AIS) are the leading causes of mortality and disability worldwide and pose significant public health challenges (1). AMI remains a primary cause of death in developed countries, while AIS ranks as the second leading cause of mortality and third leading cause of disability globally (2, 3). In 2010, Omar et al. (4) first introduced the concept of cardiocerebral infarction (CCI), defined as the simultaneous or sequential occurrence of AMI and AIS within a short timeframe.

Current CCI research faces several challenges. Firstly, the lack of consensus on the time window for AMI and AIS co-occurrence has led to widely varying reported CCI incidence rates (5), ranging from 0.009 to 12.7% (6–9). Secondly, as a complex critical syndrome involving cardiac and cerebral damage, CCI is characterized by a narrow therapeutic window, complex treatment decisions, poor prognosis, and high mortality (10). These factors significantly complicate clinical management. Therefore, a comprehensive understanding of CCI pathogenesis and clinical features is critical for developing effective treatment strategies. However, evidence-based guidelines and expert consensus on CCI management are scarce, with existing studies predominantly limited to case reports and small case series. Clinicians often rely on single-disease guidelines and empirical approaches for AMI or AIS when formulating treatment plans.

While numerous studies have independently explored predictors of short-term adverse outcomes in patients with AIS or AMI, the CCI, as a composite disease, has more complex pathophysiological mechanisms and diverse clinical features. The risk factors for adverse outcomes in CCI patients may differ significantly from those in patients with isolated AIS or AMI. However, systematic research in this area remains limited.

Given this context, our dual-center study investigated the clinical features of CCI patients and identified independent risk factors for in-hospital mortality. This study aimed to enhance the understanding of CCI clinical features and identify key prognostic factors, thereby providing an empirical foundation for advancing prevention and treatment strategies and ultimately improving clinical outcomes for CCI patients.

## 2 Materials and methods

### 2.1 Study population

We conducted a retrospective analysis of patients admitted to Zhongshan Hospital and Cardiovascular Hospital, both affiliated with Xiamen University, between January 1, 2014, and December 31, 2023. The initial cohort comprised 17,645 patients with AIS and 7,584 patients with AMI. According to the predefined inclusion and exclusion criteria, we identified 85 patients with CCI for analysis.

The inclusion criteria were: age ≥ 18 years; meeting diagnostic criteria for both AMI and AIS; simultaneous occurrence of AMI and AIS within a two-week interval (11, 12), including patients admitted with CCI or those initially diagnosed with either AMI or AIS who developed the other condition during hospitalization; and availability of complete clinical and laboratory data.

The exclusion criteria comprised: interval between AMI and AIS exceeding 2 weeks; presence of other cardiac conditions such as myocarditis or pericarditis; and incomplete clinical data.

### 2.2 Data collection

We systematically collected data from electronic medical records using a predefined structured data collection form. The collected information included demographic characteristics (sex, age), traditional cardiovascular and cerebrovascular risk factors (hypertension, diabetes, dyslipidemia, smoking and alcohol consumption history), medical history (cerebral infarction, percutaneous coronary intervention [PCI], active malignant tumor), primary symptoms, sequence of onset, and laboratory data on the day of diagnosis (routine blood tests, lipid profile, liver and renal function tests, coagulation parameters).

For AMI patients, we documented the type of AMI (ST-segment elevation myocardial infarction [STEMI] and non-ST-segment elevation myocardial infarction [NSTEMI]), baseline Global Registry of Acute Coronary Events (GRACE) score, Killip classification of cardiac function, peak myocardial injury biomarkers (high-sensitivity troponin T [hsTnT] and N-terminal pro-B-type natriuretic peptide [NT-proBNP]), echocardiography findings, and 24-h Holter electrocardiogram results.

For AIS patients, we recorded TOAST classification, occluded vessel, baseline and discharge National Institutes of Health Stroke Scale (NIHSS) scores, modified Rankin Scale (mRS) scores, relevant imaging data, and acute-phase treatment regimens, including reperfusion therapy and antiplatelet and anticoagulant therapies. The NIHSS and mRS scores at baseline were assessed by neurologists upon patient admission or during urgent consultation immediately after the onset of stroke symptoms in patients primarily admitted for myocardial infarction.

The primary endpoint was in-hospital mortality. Secondary endpoints included severe cerebrovascular adverse events (cerebral herniation and hemorrhagic transformation of cerebral infarction) and major adverse cardiovascular events (MACE), including cardiovascular death, malignant arrhythmias, cardiac rupture, ventricular septal perforation, and recurrent myocardial infarction.

The data were independently collected by specially trained researchers at each center and reviewed by another team member. Missing or anomalous data were verified through original medical record review or consultation with clinicians.

### 2.3 Definitions

We calculated the overall incidence of CCI as the ratio of CCI cases to the total number of AIS and AMI cases. CCI were classified into two types based on the order of onset: synchronous and metachronous (6). Synchronous CCI was defined as the simultaneous presentation of acute focal neurological deficits (confirmed as AIS by imaging studies) and evidence of AMI at admission. AMI evidence included significant elevations in cardiac biomarkers, accompanied by ischemic electrocardiographic changes and/or typical clinical symptoms. Metachronous CCI was defined as the diagnosis of either AMI or AIS at admission, followed by the occurrence of the other condition during hospitalization.

Hemorrhagic transformation (HT) of cerebral infarction was classified based on the European Cooperative Acute Stroke Study (ECASS) criteria. Hemorrhagic infarction type 1 (HI1) was defined as small petechial hemorrhages along the margins of the infarct region. Hemorrhagic infarction type 2 (HI2) was characterized by more confluent petechiae within the infarct area, without space-occupying effect. Parenchymal hematoma type 1 (PH1) was defined as hematoma involving ≤ 30% of the infarct area with some mild space-occupying effect, while parenchymal hematoma type 2 (PH2) represented hematoma involving > 30% of the infarct area with significant space-occupying effect or hemorrhage distant from the infarct area. Malignant cerebral edema was defined as a midline shift ≥ 5mm on neuroimaging.

We assessed short-term neurological prognosis using the mRS, with a score ≤ 2 defined as functional independence. The neutrophil-to-lymphocyte ratio (NLR) was calculated by dividing the absolute neutrophil count by the absolute lymphocyte count in peripheral blood. An active malignant tumor was defined as a malignancy initially diagnosed within 6 months prior to CCI onset or during hospitalization, or evidence of recurrence, metastasis, or progression of a preexisting malignancy (13).

### 2.4 Statistical analysis

Statistical analyses were performed using SPSS 26.0 software (IBM, Chicago, USA). The Shapiro–Wilk test was used to assess the normality of continuous variable distribution. Normally distributed continuous variables are reported as means ± standard deviations and compared using independent samples t tests. Non-normally distributed continuous variables are presented as medians with interquartile ranges (IQR) and compared using Mann–Whitney U tests. Categorical variables are expressed as frequencies and percentages [*n* (%)] and analyzed using chi-square tests or Fisher’s exact tests, as appropriate.

To examine correlations between variables and identify potential mediators, we conducted Spearman correlation analysis. The correlation coefficient (*r*) values were interpreted as: weak (0.00–0.39), moderate (0.40–0.59), strong (0.60–0.79), and very strong (0.80–1.00). Variables with strong correlations (|r| > 0.6) were screened to exclude mediating variables and avoid multicollinearity.

Selected variables underwent multivariate logistic regression analysis to identify independent predictors. For statistically significant continuous variables (*p* < 0.05) in the multivariate analysis, we plotted receiver operating characteristic (ROC) curves and calculated the area under the curve (AUC) with 95% confidence intervals (CI). Youden’s index was used to determine optimal cutoff values, sensitivity, and specificity. All statistical tests were two-sided, with *p* < 0.05 considered to indicate statistical significance.

## 3 Results

### 3.1 Clinical features

Our study enrolled 85 CCI patients, comprising 64.71% males (*n* = 55) and 35.29% females (*n* = 30), with a median age of 71 years (IQR: 64–78). Regarding lifestyle factors, long-term smoking history was reported in 42.35% of patients, while 14.12% reported alcohol consumption. Prevalent comorbidities included hypertension (70.59%), atrial fibrillation (38.82%), hyperlipidemia (31.26%), and diabetes (30.59%). Notably, 7.10% of patients (*n* = 6) presented with active malignancies.

The temporal sequence of CCI events varied: 45.88% (*n* = 39) of patients developed AIS subsequent to admission for AMI, 31.76% (*n* = 27) experienced AMI following admission for AIS, and 22.35% (*n* = 19) presented with simultaneous AIS and AMI.

A total of 42 patients (49.41%) sought medical attention due to AMI symptoms, of whom STEMI accounted for 56.47%. The median baseline GRACE score was 163.00 (IQR: 130.50–179.00), with 29.41% (*n* = 25) of patients categorized as Killip class III-IV. Echocardiography revealed ventricular wall motion abnormalities in 82.93% of patients, ventricular tumors in 15.85%, valvular disease in 28.05%, and left ventricular thrombus in 10.98%.

For patients who initially presented with AIS symptoms (50.59%, *n* = 43), the median NIHSS score was 7.00 (IQR: 4.00–14.25), with 42.35% of patients demonstrating minimal to moderate disability (mRS 0–2). Etiological analysis revealed that cardioembolism as the predominant subtype (52.94%), followed by large artery atherosclerosis (29.41%). Hypercoagulable states associated with active malignancies accounted for 5.88% of cases, with a single case (1.17%) attributed to aortic dissection. AIS lesions primarily affected the anterior circulation, showing a slight predilection for the right hemisphere (54.12% *vs* 50.00% left), while the vertebrobasilar system was involved in 15.29% of patients (Table 1).

**Table 1:** Baseline characteristics and clinical features of patients with concurrent acute myocardial infarction and stroke.

	Total (*n* = 85)	In-hospital mortality (*n* = 26)	Discharge alive (*n* = 59)	*p*-value
Age, year	71.00 (64.00, 78.00)	76.50 (65.00, 80.00)	71.00 (62.50, 77.50)	0.158
Sex, male	55 (64.71)	14 (53.85)	41 (69.49)	0.219
Smoking	36 (42.35)	10 (38.46)	26 (44.07)	0.644
Alcohol	12 (14.12)	4 (15.38)	8 (13.56)	>0.99
Initial symptom, AMI	42 (49.41)	14 (53.85)	29 (49.15)	0.815
Order of infarction				0.273
Cardiac-cerebral	39 (45.88)	13 (50.00)	26 (44.07)	
Cerebral-cardiac	27 (31.76)	10 (38.46)	17 (28.81)	
Concurrent	19 (22.35)	3 (11.54)	16 (27.12)	
Past medical history				
Diabetes	26 (30.59)	7 (26.92)	19 (32.20)	0.799
Hypertension	60 (70.59)	17 (65.38)	43 (72.88)	0.606
Dyslipidemia	27 (31.76)	6 (23.08)	21 (35.59)	0.317
History of PCI	13 (15.29)	6 (23.08)	7 (11.86)	0.204
History of stroke	17 (20.00)	4 (15.38)	13 (22.03)	0.568
Atrial fibrillation	33 (38.82)	13 (50.00)	20 (33.90)	0.227
Active malignant tumor	6 (7.10)	4 (15.4)	2 (3.39)	0.068
Clinical feature of AMI				
Type of AMI, STEMI	48 (56.47)	20 (76.92)	28 (47.46)	**0.017**
Killip III ~ IV	25 (29.41)	18 (69.20)	6 (10.20)	**<0.001**
LV thrombus	9 (10.98)	3 (13.04)	6 (10.17)	0.750
Valvular disease	23 (28.05)	8 (34.78)	15 (25.42)	0.422
Ventricular aneurysm	13 (15.85)	5 (21.74)	8 (13.56)	0.501
Regional wall abnormalities	68 (82.93)	18 (78.26)	50 (84.75)	0.522
Baseline GRACE score	161.00 (131.00, 179.00)	194.00 (175.00, 224.00)	150.50 (124.00, 168.50)	**<0.001**
MACE	24 (28.24)	20 (76.92)	4 (6.78)	**<0.001**
Clinical feature of AIS				
TOAST classification				0.099
Large-artery atherosclerosis	25 (29.41)	7 (26.92)	18 (30.51)	
Cardioembolism	45 (52.94)	14 (53.85)	31 (52.54)	
Small-artery occlusion	7 (8.24)	0 (0.00)	7 (11.86)	
Other determined etiology	2 (2.35)	1 (3.85)	1 (1.69)	
Undetermined etiology	6 (7.06)	4 (15.38)	2 (3.39)	
Occluded vessel				
Left anterior circulation	42 (50.00)	13 (50.00)	29 (50.00)	>0.99
Right anterior circulation	46 (54.12)	13 (50.00)	33 (55.93)	0.613
Vertebrobasilar artery	13 (15.29)	5 (19.23)	8 (13.56)	0.525
Baseline NIHSS score	7.00 (4.00, 15.00)	16.00 (12.00, 27.00)	5.00 (2.00, 9.50)	**<0.001**
Baseline mRS score	3.00 (1.00, 5.00)	5.00 (4.75, 5.00)	2.00 (1.00, 4.00)	**<0.001**
Brain herniation	4 (4.71)	3 (11.54)	1 (1.69)	0.083
Hemorrhagic transformation	7 (8.24)	3 (11.54)	4 (6.78)	0.670

AMI, acute myocardial infarction; PCI, percutaneous coronary intervention; STEMI, ST-elevation myocardial infarction; LV, left ventricular; GRACE, Global Registry of Acute Coronary Events; MACE, major adverse cardiac events; AIS, acute ischemic stroke, NIHSS, National Institutes of Health Stroke Scale; mRS, modified Rankin Scale.

Furthermore, among CCI patients presenting with myocardial infarction as the initial manifestation (49.41%, *n* = 42), 26 cases (30.59%) were classified as cardiogenic embolism according to the TOAST classification. Of these, 6 cases were attributed to cerebral infarction following cardiac interventional procedures, 16 cases were due to cerebral embolism caused by atrial fibrillation, 3 cases resulted from cerebral infarction secondary to left ventricular thrombus dislodgement, and 1 case was caused by the combination of atrial fibrillation and left ventricular thrombus.

### 3.2 Incidence and outcomes of adverse events

Cerebrovascular complications were observed in 15.29% of patients, including hemorrhagic transformation of cerebral infarction (8.24%, *n* = 7) and malignant cerebral edema (7.06%, *n* = 6), with the latter leading to cerebral herniation in 4 patients (4.71%). Among the 7 patients with hemorrhagic transformation, the specific subtypes were as follows: 2 patients with HI1, 1 patient with HI2, and 4 patients with PH1.

The in-hospital mortality rate was notably high at 30.59% (26/85), with cardiovascular causes accounting for the majority (65.38%) of fatalities. Other causes of death included cerebral herniation (7.69%, *n* = 2), multiple organ failure, respiratory failure, and septic shock (3.85% each, *n* = 1). In 15.38% (*n* = 4) of the patients, the precise cause of death remained undetermined. Among survivors (69.41%, *n* = 59), a significant proportion (66%, *n* = 39) achieved neurological independence (mRs ≤ 2) at discharge (Table 1).

### 3.3 Determinants of in-hospital mortality

Univariate analysis comparing CCI patients who died in-hospital (*n* = 26) with survivors (*n* = 59) revealed several significant predictors of mortality. However, demographic characteristics, traditional vascular risk factors, medical history, initial symptoms, and sequence of onset showed no significant associations (all *p* > 0.05), and STEMI incidence (76.92% *vs* 47.46%, *p* = 0.012) and higher Killip classification and GRACE scores (both *p* < 0.001) emerged as significant AMI-related predictors (Table 1).

For AIS patients, higher baseline NIHSS and mRS scores (both *p* < 0.001) strongly correlated with mortality. Laboratory analysis revealed a more pronounced inflammatory response in the death group, as evidenced by significantly increased white blood cell counts, neutrophil counts, and NLR values (all *p* < 0.001), as well as significantly lower lymphocyte counts (*p* = 0.005). The D-dimer, peak hsTnT and NT-proBNP levels were also significantly greater in the nonsurviving group (all *p* < 0.001). Furthermore, hemoglobin and serum albumin levels were significantly lower in the nonsurviving group (both *p* = 0.002) (Table 2).

**Table 2:** Comparison of treatment strategies between patients with in-hospital mortality and discharged alive.

Variables	Total (*n* = 85)	In-hospital mortality (*n* = 26)	Discharge alive (*n* = 59)	*p*-value
Acute reperfusion therapy				
Intravenous thrombolysis	7 (8.24)	3 (11.54)	4 (6.78)	0.670
Mechanical thrombectomy	6 (7.06)	3 (11.54)	3 (5.08)	0.541
Percutaneous coronary intervention	45 (52.94)	10 (38.46)	35 (59.32)	0.062
Antiplatelet therapy strategy				**0.014**
No antiplatelet therapy	9 (10.59)	5 (19.23)	4 (6.78)	
Single antiplatelet therapy	21 (24.71)	10 (38.46)	11 (18.64)	
Dual antiplatelet therapy	55 (64.71)	11 (42.31)	44 (74.58)	
Anticoagulation therapy	44 (51.76)	18 (69.23)	26 (44.07)	**0.037**

Bold values indicate statistical significance (*p* < 0.05).

### 3.4 Interrelationships between key clinical variables

**Figure 1:**
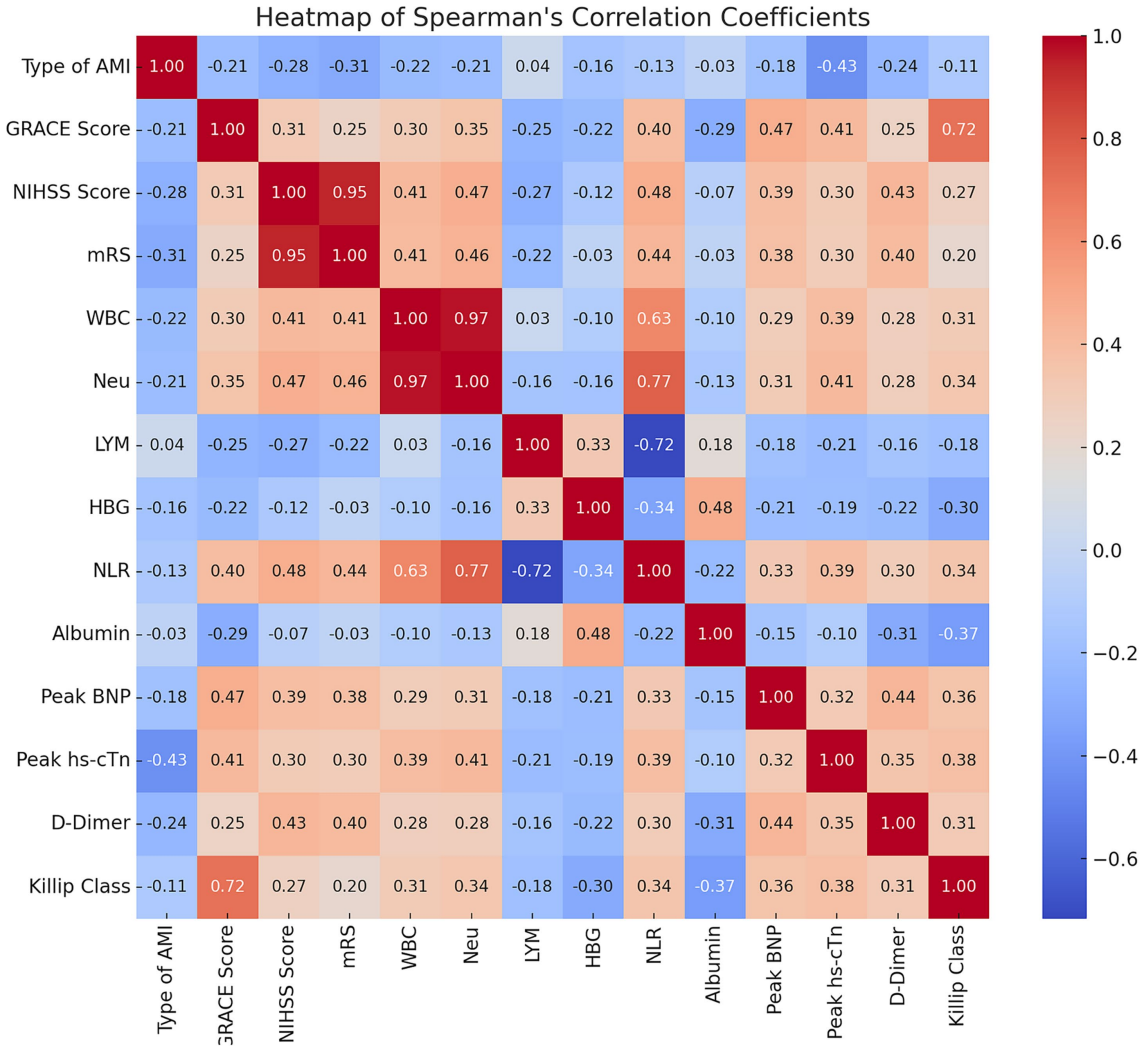
Heatmap of Spearman’s correlation coefficients among key clinical variables in CCI patients. Heatmap showing Spearman’s correlation coefficients between clinical variables in cardiocerebral infarction (CCI) patients. Color intensity and numbers indicate correlation strength and coefficient. Red: positive correlations; Blue: negative correlations. Strong correlations: NLR with WBC (*r* = 0.63) and neutrophils (*r* = 0.77), GRACE score with Killip class (*r* = 0.72), NIHSS with initial mRS (*r* = 0.95).

Spearman correlation analysis revealed significant interrelationships among critical clinical variables in CCI patients. The NLR emerged as a central inflammatory marker, demonstrating moderate to strong positive correlations with white blood cell count (*r* = 0.633, *p* < 0.001) and neutrophil count (*r* = 0.774, *p* < 0.001) and a strong negative correlation with lymphocyte count (*r* = −0.717, *p* < 0.001). Additionally, the GRACE score at admission showed a strong positive correlation with the Killip classification (*r* = 0.717, *p* < 0.001), and the NIHSS score at admission correlated strongly with the initial mRS score (*r* = 0.946, *p* < 0.001) (Figure 1).

Considering that the NLR is derived from neutrophil and lymphocyte counts, and that the GRACE score incorporates the Killip classification, we opted to retain the GRACE score, baseline NIHSS score, and NLR as independent variables in subsequent analyses, while excluding other related factors based on these correlation results.

### 3.5 Predictive model for adverse outcomes

**Figure 2:**
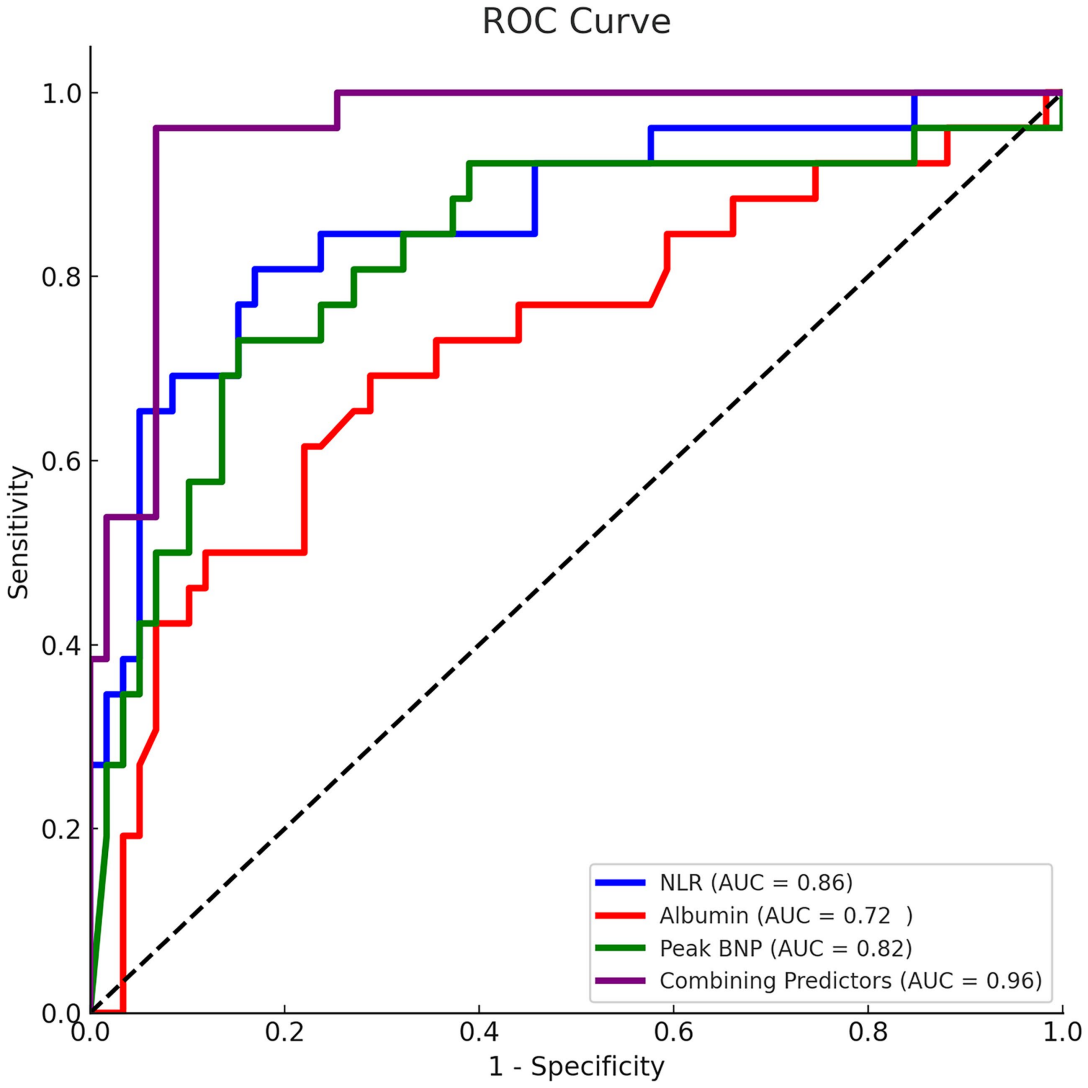
ROC curves for predicting in-hospital mortality in CCI patients.

Multivariate logistic regression analysis was performed using nine variables selected based on their statistical significance (*p* < 0.05) in univariate analysis after excluding mediating variables. These included the type of AMI, initial GRACE score, baseline NIHSS score, hemoglobin, NLR, albumin, peak NT-proBNP, peak hsTnT and D-dimer levels. The regression analysis identified three independent risk factors for in-hospital mortality: increased NLR (OR = 1.400, 95% CI: 1.053–1.860, *p* = 0.020), low albumin levels (OR = 0.695, 95% CI: 0.486–0.995, *p* = 0.047), and high peak NT-proBNP levels (OR = 1.000, 95% CI: 1.000–1.000, *p* = 0.045) (Table 3; Figure 2).

**Table 3:** Comparison of laboratory parameters between patients with in-hospital mortality and those discharged alive.

Variables	Total (*n* = 85)	In-hospital mortality (*n* = 26)	Discharge alive (*n* = 59)	*p*-value
White blood cell	9.86 (8.17, 14.65)	15.50 (12.34, 17.77)	8.95 (7.36, 11.74)	**<0.001**
Neutrophils	7.86 (5.70, 12.17)	13.04 (9.75, 15.39)	6.13 (4.70, 8.79)	**<0.001**
Lymphocytes	1.43 (1.00, 2.06)	1.08 (0.77, 1.67)	1.54 (1.20, 2.09)	**0.005**
Hemoglobin	116.27 ± 27.49	102.77 ± 25.08	122.22 ± 26.56	**0.002**
Platelets	227.00 (166.00, 309.00)	227.50 (140.0, 309.0)	222.00 (166.0, 313.5)	0.808
NLR	5.87 (3.02, 10.72)	12.59 (7.70, 18.55)	3.81 (2.67, 6.41)	**<0.001**
Total protein	65.91 ± 7.89	65.14 ± 8.99	66.25 ± 7.42	0.553
Albumin	35.12 ± 4.75	32.74 ± 4.67	36.17 ± 4.42	**0.002**
Triglycerides	1.25 (0.99, 1.67)	1.20 (0.88, 1.67)	1.27 (0.99, 1.65)	0.564
Total cholesterol	4.43 ± 1.37	4.24 ± 1.22	4.51 ± 1.43	0.397
High density lipoprotein	0.93 (0.83, 1.16)	0.93 (0.86, 1.16)	0.96 (0.82, 1.17)	0.815
Low density lipoprotein	2.98 ± 1.06	2.84 ± 0.99	3.05 ± 1.10	0.415
Creatinine	99.20 (69.20, 125.70)	113.20 (82.20, 162.20)	90.40 (66.10, 119.50)	0.083
Uric acid	376.54 ± 140.46	337.53 ± 164.81	393.73 ± 126.03	0.089
D-dimer	2.05 (1.02, 4.81)	5.16 (2.54, 14.64)	1.42 (0.90, 3.25)	**<0.001**
Fibrinogen	4.29 (3.37, 5.61)	4.68 (3.43, 6.53)	4.23 (3.35, 5.49)	0.203
Peak hs-cTn	3399.0(1045.0, 6057.0)	6057.0 (3418.3, 8623.0)	2433.0 (638.50, 4277.0)	**<0.001**
Peak NT-proBNP	5826.0(2343.0, 13,179)	14856.5 (7866.0, 33555.0)	3790.0 (2084.5, 7301.0)	**<0.001**

NLR, Neutrophil-to-Lymphocyte Ratio; NT-proBNP, brain natriuretic.

Reference ranges: WBC: 3.5–9.5 × 10^9/L, neutrophils: 1.8–6.3 × 10^9/L, lymphocytes: 1.1–3.2 × 10^9/L, hemoglobin: 13.0–17.5 g/dL, platelets: 125–350 × 10^9/L, Total protein: 65–85 g/L, albumin: 40–55 g/dL, triglycerides: <1.7 mg/dL, Total cholesterol:3.0–5.7 mmol/L, HDL-C: >1.04 mmol/L, LDL-C: <4.21 mmol/L, creatinine: 57–111 umol/L, uric acid: 208–428 uumol/L, D-dimer: <5.5 mg/L, fibrinogen: 2.0–4.0 g/L, peak cTnT: <14 ng/L, peak NT-proBNP: <125 pg/mL.

Bold values indicate statistical significance (*p* < 0.05).

ROC curve analysis revealed strong predictive performance for these factors individually with AUC for NLR, albumin, and peak NT-proBNP were 0.863 (95% CI: 0.773–0.954), 0.723 (95% CI: 0.599–0.847), and 0.824 (95% CI: 0.718–0.930), respectively (all *p* ≤ 0.01). The optimal cutoff values were 6.914 for NLR (sensitivity 80.8%, specificity 83.1%), 33.80 g/L for albumin (sensitivity 69.2%, specificity 71.2%), and 9474.5 pg/mL for peak NT-proBNP (sensitivity 73.1%, specificity 84.7%). Notably, a combined predictive model incorporating these three markers demonstrated superior performance (AUC = 0.959, 95% CI: 0.920–0.998, *p* < 0.01; sensitivity 96.2%, specificity 93.2%), significantly outperforming individual indicators (Table 4).

**Table 4:** Multivariable logistic regression for mortality prediction in CCI patients.

Variables	Regression coefficient	standard error	Wald *χ*2	OR (95% CI)	*p*-value
Type of AMI	−3.834	2.005	3.657	0.022 (0.000, 1.100)	0.056
Initial GRACE score	0.020	0.012	2.456	1.020 (0.995, 1.045)	0.117
Initial NIHSS score	0.088	0.083	1.120	1.092 (0.927, 1.287)	0.290
Hemoglobin	−0.006	0.021	0.072	0.994 (0.955, 1.036)	0.788
NLR	0.336	0.145	5.372	1.400 (1.053, 1.860)	**0.020**
Albumin	−0.363	0.183	3.956	0.695 (0.486, 0.995)	**0.047**
Peak NT-proBNP	0.000	0.000	4.007	1.000 (1.000, 1.000)	**0.045**
Peak hs-cTn	0.000	0.000	0.427	1.000 (1.000, 1.000)	0.513
D-dimer	0.092	0.139	0.440	1.097 (0.835, 1.440)	0.507

AMI, acute myocardial infarction; NLR, neutrophil-to-lymphocyte ratio; NT-proBNP, N-terminal pro-B-type natriuretic peptide; hs-cTn, high-sensitivity cardiac troponin; GRACE, Global Registry of Acute Coronary Events. Bold values indicate statistical significance (*p* < 0.05).

### 3.6 Therapeutic strategies and efficacy

For reperfusion therapy, intravenous thrombolysis (AIS, 0.9mg/kg) and mechanical thrombectomy were employed in 8.24% (*n* = 7) and 7.06% (*n* = 6) of patients, respectively, with no significant difference in utilization between survival groups (*p* > 0.05). However, a trend toward higher rates of PCI was observed in survivors than in nonsurvivors (59.32% *vs* 38.46%, *p* = 0.076).

Regarding medication, dual antiplatelet therapy (DAPT) was significantly more common among survivors than among deceased patients (74.58% vs. 42.31%, *p* = 0.014). Conversely, the proportion of patients who did not receive any antiplatelet therapy was significantly greater in the nonsurviving group (19.23% vs. 6.78%, *p* = 0.014). Furthermore, anticoagulant therapy was also used significantly more frequently in the nonsurviving group (69.23% vs. 44.07%, *p* = 0.037) (Table 5).

**Table 5:** ROC Analysis of laboratory parameters for predicting mortality in CCI patients.

Variables	AUC	Youden index J	Sensitivity	Specificity	Cut-off Value	95% CI	*p*-value
NLR	0.863	0.639	0.808	0.831	6.914	0.773–0.954	**<0.001**
Albumin (g/L)	0.723	0.404	0.692	0.712	33.80	0.599–0.847	**0.001**
Peak NT-proBNP (pg/mL)	0.824	0.578	0.731	0.847	9474.50	0.718–0.930	**<0.001**
Combined Diagnosis	0.959	0.894	0.962	0.932	0.300	0.920–0.998	**<0.001**

NLR, Neutrophil-to-Lymphocyte Ratio; NT-proBNP, N-terminal pro-B-type natriuretic peptide. Bold values indicate statistical significance (*p* < 0.05).

## 4 Discussion

### 4.1 Distinct clinical profile of CCI

Our study revealed an overall CCI incidence of approximately 0.34%, with male patients comprising approximately twice the proportion of female patients. CCI patients exhibited risk factors similar to those associated with traditional cardiovascular and cerebrovascular diseases, including hypertension, diabetes, dyslipidemia, long-term smoking, and alcohol consumption. These findings align closely with a previous meta-analysis by Ng et al. (14), which reported that 65.9% of CCI patients were male, with a majority having a long-term smoking history (27.3%) and comorbidities including hypertension (31.8%), diabetes mellitus (15.9%), and dyslipidemia (11.4%). These common risk factors contribute to atherosclerosis development. Atherosclerotic plaque rupture triggers platelet activation and thrombus formation, which may sequentially or simultaneously affect the coronary arteries and the internal carotid artery/vertebrobasilar artery, leading to the development of AMI and AIS (15).

Our study further found several specific risk factors for CCI, including atrial fibrillation, left ventricular thrombus formation, active malignant neoplasms associated with a systemic hypercoagulable state, and aortic dissection. Each of these factors increases CCI risk through distinct mechanisms. For instance, atrial fibrillation can lead to the dislodgement of thrombi from the atrium, potentially causing cerebral and coronary embolisms (16). AMI affecting the apex and anterior wall may increase the likelihood of mural thrombus formation due to cardiac pump failure and reduced ejection fraction. Aortic dissection originating in the ascending aorta can result in an intimal tear. When this tear extends longitudinally to involve the coronary ostia and the carotid or vertebrobasilar arteries, it may also precipitate CCI (15, 17).

Regarding clinical manifestations, the initial symptoms of CCI patients are often nonspecific, with approximately half seeking medical attention due to typical symptoms of either AMI or AIS. STEMI was the most common type of AMI in our cohort, consistent with previous findings (7). These patients frequently present with comorbidities such as ventricular wall motion abnormalities, ventricular thrombus formation, and valvular disease. In CCI patients, the etiology of AIS is predominantly cardiogenic cerebral embolism, with lesions primarily occurring in the right anterior circulation. Notably, despite experiencing concurrent AMI and AIS, approximately half of the CCI patients in our study exhibited good neurological status upon admission (mRS ≤ 2), with a median NIHSS score of 7.00 (IQR: 4.00, 14.25). This suggests that AMI complications may not significantly exacerbate AIS severity in CCI patients. These findings align with previous research reporting that 78.6% of CCI patients experienced STEMI, and 54.2% had NIHSS scores between 5 and 15 points, indicating moderate neurological impairment (14).

However, the mortality risk in CCI patients remains notably high, with approximately one-third of our cohort succumbing during hospitalization. This finding aligns with previously reported mortality rates ranging from 21.4 to 45% (18, 19). Cardiovascular events were the predominant cause of death, consistent with earlier findings (14).

Our analysis revealed a greater proportion of survivors among CCI patients who underwent PCI than among those who died in the hospital (59.32% vs. 38.46%, *p* = 0.076). Although this difference did not reach statistical significance, it aligns with findings of a large-scale study by Alqahtani et al. Their analysis of 13,573 CCI patients reported that PCI was associated with significantly reduced in-hospital mortality (OR = 0.26, 95% CI: 0.20–0.34, *p* < 0.001). These findings collectively suggest a potentially crucial role for the PCI in improving the prognosis of patients with CCI.

The pharmacological management of CCI patients, particularly those with atrial fibrillation, presents significant challenges. While anticoagulation is essential for preventing thrombus formation in atrial fibrillation patients, it markedly increases the risk of hemorrhagic transformation in those with extensive cerebral infarction. Furthermore, the decision between DAPT for AMI and single antiplatelet therapy for stroke adds complexity to treatment strategies. Our study revealed significantly greater DAPT usage among survivors than among deceased patients. Conversely, the majority of those who died had received either no antiplatelet therapy or single antiplatelet therapy. However, it is crucial to interpret this association cautiously. Rather than directly implying a prognostic improvement due to DAPT, this observation may reflect a ‘healthy survivor bias’. Patients in the mortality group, potentially with more severe or extensive infarctions, higher bleeding risks, or multiple comorbidities, might have prompted clinicians to opt for more conservative treatment approaches.

### 4.2 Predictors of in-hospital mortality in CCI

Our study identified increased NLR, decreased serum albumin and elevated peak NT-proBNP levels as independent risk factors for in-hospital mortality in CCI patients. The NLR, a composite indicator of neutrophil and lymphocyte counts, has been consistently associated with short-term adverse outcomes in both AMI and AIS patients in previous studies (20, 21). However, the mechanisms underlying the predictive value of the NLR may differ between these conditions.

In Type I myocardial infarction, the most common and clinically significant form of AMI, neutrophils play a complex and critical role. This condition is typically precipitated by the rupture of intracoronary thin-cap fibroatheromas (TCFA) (12). Neutrophils can promote TCFA rupture through the release of neutrophil extracellular traps, thereby triggering AMI. Following infarction, necrotic myocardial tissue releases damage-associated molecular patterns (DAMP), which bind to Toll-like receptors on neutrophil surfaces. This interaction initiates a cascade of reactions resulting in the production and activation of proinflammatory (N1-type) neutrophils (22). These activated neutrophils subsequently release various inflammatory cytokines and reactive oxygen species (ROS), further exacerbating myocardial injury in the infarcted area (22).

Concurrently, the systemic stress response induced by myocardial infarction significantly affects lymphocyte populations. Stress-induced activation of the neuroendocrine system leads to increased levels of catecholamines and glucocorticoids. These hormones promote lymphocyte apoptosis and inhibit lymphocyte proliferation and differentiation, ultimately resulting in decreased peripheral blood lymphocyte counts (23, 24).

In AIS, inflammatory processes are pivotal in disease progression. Damaged brain cells release inflammatory factors, chemokines, and neurotoxic substances, which disrupt the blood–brain barrier (25). This triggers an inflammatory cascade, inducing neutrophil and immune cell migration into brain tissue, mediating secondary neuronal injury, and exacerbating neurological dysfunction (26). Furthermore, these infiltrating neutrophils further damage ischemic tissues by releasing proinflammatory mediators, proteases, and matrix metalloproteinases (27). Conversely, certain lymphocyte subsets, such as regulatory T cells, may improve patient prognosis by suppressing excessive inflammation and promoting neuronal repair (28). The NLR provides a more comprehensive and stable reflection of a patient’s inflammatory status, oxidative stress levels, and degree of immune imbalance compared to individual neutrophil or lymphocyte counts. In CCI, where both cardiac and cerebral tissues are affected, the NLR captures the cumulative impact of these complex inflammatory processes. This multifaceted representation renders the NLR a powerful predictor, capable of accurately assessing the risk of in-hospital mortality in CCI patients.

Serum albumin, a multifunctional protein that serves as a crucial clinical marker, plays diverse physiological roles (29). At physiological concentrations, albumin exerts anti-inflammatory effects on endothelial cells (30). However, reduced albumin levels are typically associated with malnutrition and inflammatory conditions (31), triggering a cascade of detrimental effects, including increased oxidative stress, enhanced platelet activation and aggregation, and elevated thrombotic risk (32–34). Moreover, as a major plasma protein and drug-binding agent, albumin critically influences drug distribution and metabolism; thus, hypoalbuminemia may alter hemorheological properties and therapeutic efficacy (35). The cumulative impact of these factors can significantly impair patients’ resilience to acute events, increase complication risks, and ultimately affect survival rates. Previous studies have consistently demonstrated a strong association between reduced serum albumin levels and adverse outcomes in AIS and AMI patients (35, 36).

Elevated NT-proBNP levels typically reflect increased ventricular wall tension and the extent of ventricular remodeling (37). In CCI patients, elevated NT-proBNP often correlates with excessive activation of the renin-angiotensin-aldosterone system and the sympathetic nervous system (38). This persistent neuroendocrine activation leads to vasoconstriction, sodium and water retention, myocardial fibrosis, and cardiac dysfunction, creating a detrimental cycle of worsening cardiac function (39). Furthermore, brain injury, such as AIS, can further stimulate NT-proBNP release through the neuroendocrine-immune axis (40), potentially exacerbating neurological impairment (41). Consequently, NT-proBNP has emerged not only as a key indicator for assessing cardiac functional status but also as a potential marker reflecting cerebral functional status.

This study has several noteworthy limitations. Our focus on in-hospital mortality without long-term follow-up data precludes a comprehensive evaluation of CCI patients’ long-term prognosis. Despite accounting for numerous known confounding factors, the influence of unidentified or inadequately measured variables cannot be entirely ruled out. Secondly, it is worth contemplating the frequency of embolism into the coronary arteries in cases of cardiogenic cerebral embolism and whether coronary plaque rupture was considered as atherosclerotic disease after non-cardiogenic cerebral infarction. Investigating these aspects would contribute to a deeper understanding of the pathogenic mechanisms underlying CCI. However, due to the retrospective nature of the study and the lack of complete cardiac imaging and coronary angiography data, we were unable to provide a detailed analysis of these factors. Future prospective studies should incorporate thorough cardiac examinations to elucidate the specific mechanisms of myocardial infarction following different subtypes of cerebral infarction. Furthermore, the retrospective design of the study and the insidious onset of myocardial infarction posed significant challenges in precisely determining the time interval between the onset of cerebral and myocardial infarction in some patients. For cases of concurrent myocardial and cerebral infarction at admission, establishing the exact sequence and time interval between the two events was difficult. This limitation may affect our understanding of the temporal and causal relationships between myocardial and cerebral infarction. To address these limitations and strengthen our findings, future research should employ a multicenter, prospective approach with a larger sample size and extended follow-up duration. Incorporating a broader array of clinical, biochemical, and imaging indicators, along with serial biomarker measurements, would provide a more holistic view of CCI pathophysiology and elucidate potential prognostic implications. Ultimately, such improvements will aid in more precisely assessing the prognostic factors for CCI patients and provide a stronger scientific foundation for clinical decision-making.

## 5 Conclusion

Our analysis of CCI revealed that the incidence of CCI is approximately 0.34%, with males being approximately twice as likely as females to develop this condition. In CCI patients, AMI primarily manifests as STEMI, while AIS is predominantly of cardiogenic origin. Approximately one-third of CCI patients succumb during hospitalization, with cardiovascular events emerging as the primary cause of mortality. We identified three independent risk factors for in-hospital mortality: elevated neutrophil-to-lymphocyte ratio, decreased serum albumin, and increased peak NT-proBNP levels. These biomarkers, individually or in combination, demonstrated robust predictive capability for adverse outcomes. Our study significantly enhances our understanding of CCI, delineates key prognostic factors, and identifies potential therapeutic targets to improve clinical outcomes in this unique patient population.

## Data Availability

The raw data supporting the conclusions of this article will be made available by the authors, without undue reservation.
